# On Genetic Specificity in Symbiont-Mediated Host-Parasite Coevolution

**DOI:** 10.1371/journal.pcbi.1002633

**Published:** 2012-08-30

**Authors:** Marek Kwiatkowski, Jan Engelstädter, Christoph Vorburger

**Affiliations:** 1 Institute of Integrative Biology, ETH Zürich, Zürich, Switzerland; 2 Eawag: Swiss Federal Institute of Aquatic Science and Technology, Dübendorf, Switzerland; 3 Institute of Biogeochemistry and Pollutant Dynamics, ETH Zürich, Zürich, Switzerland; 4 School of Biological Sciences, The University of Queensland, Brisbane, Queensland, Australia; Pennsylvania State University, United States of America

## Abstract

Existing theory of host-parasite interactions has identified the genetic specificity of interaction as a key variable affecting the outcome of coevolution. The Matching Alleles (MA) and Gene For Gene (GFG) models have been extensively studied as the canonical examples of specific and non-specific interaction. The generality of these models has recently been challenged by uncovering real-world host-parasite systems exhibiting specificity patterns that fit neither MA nor GFG, and by the discovery of symbiotic bacteria protecting insect hosts against parasites. In the present paper we address both challenges, simulating a large number of non-canonical models of host-parasite interactions that explicitly incorporate symbiont-based host resistance. To assess the genetic specialisation in these hybrid models, we develop a quantitative index of specificity applicable to any coevolutionary model based on a fitness matrix. We find qualitative and quantitative effects of host-parasite and symbiont-parasite specificities on genotype frequency dynamics, allele survival, and mean host and parasite fitnesses.

## Author Summary

Coevolution between hosts and parasites is believed to be central to a number of biological phenomena, most notably the observed patterns of biodiversity and the origins of sexual reproduction. However, classical mathematical models of host-parasite coevolution account neither for the hosts' use of bacterial symbionts for protection from parasites, nor for the potential and observed complexity of genetic interactions between the coevolving species. In this article we address both challenges by simulating a large number of models of host-symbiont-parasite coevolution based on randomly generated genotype interaction patterns. We demonstrate that the degree of “specificity” between the genotypes of the interacting species is a major factor influencing the outcome of coevolution. We also observe that the symbionts may take over from the hosts the coevolutionary arms race against the parasites. Overall, our results make clear that the complex interaction patterns and the defensive symbionts can both play vital roles in host-parasite coevolution. An additional contribution of the article is a numerical index of specificity, applicable to a wide range of existing and future coevolutionary models.

## Introduction

Parasitism is one of the main lifestyles in nature and a major source of evolutionary pressure. Despite its central place in evolutionary ecology, however, the details of the genetic architecture underlying resistance and infectivity are not known for most host-parasite associations. Mathematical models of host-parasite coevolution compensate for the missing data by making explicit, first-principle assumptions about the interaction of host and parasite genotypes. Two such classic assumptions, and consequently two classic families of models, are known as Matching Alleles (MA) and Gene For Gene (GFG). The MA models, inspired by vertebrate immune systems [1], assume that an exact, lock-and-key match between host and parasite genotypes is required for successful infection. The GFG models, based on studies of plant disease [2], [3], postulate that an infection takes place if every “resistance” allele of the host is countered by a “virulence” allele of the parasite. Perhaps the farthest-reaching difference between the two is in the genetic specificity of the interaction. Under MA parasites exhibit full genetic specialisation to their host: a single parasite genotype can only infect a single host genotype. Under GFG, on the other hand, the number of host genotypes that a parasite may infect depends of the number of virulence alleles it has, and ranges from one genotype to all. The perfect specificity of MA interactions readily results in negative frequency-dependent selection and persistent cyclic dynamics of genotype frequencies in hosts and parasites (“Red Queen dynamics”), which in turn underpin the Red Queen Hypothesis (RQH) for the evolution of sexual reproduction [4], [5]. In contrast, in the GFG models the parasite carrying all virulence alleles takes over the population, at least until costs of infectivity and resistance are assumed [3], [6].

One problem with the existing theory is that there is a mounting number of natural systems for which the interactions between host and parasite genotypes have been disentangled and found to be neither of the MA nor the GFG kind [7]–[10]. Coupled with the uncertainty as to the extent to which plant disease data supports the GFG model [3], [11], these findings cast doubt on the generality and explanatory power of interaction models as simple as MA or GFG. Agrawal and Lively [12] tackled this problem by considering a range of non-standard genetic interactions spanning a particular continuum between MA and GFG, and found that MA-like behaviour, in particular Red Queen dynamics, is sufficiently common among non-standard models to support the generality of the RQH. Their work has since been generalised by Engelstädter and Bonhoeffer [13], who sampled a much broader range of interaction patterns. While they were able to confirm the pervasiveness of Red Queen dynamics, they also found effects that are entirely absent from the MA and GFG models, showing that non-standard models should not be ignored.

We aim to build on these two studies to address a new challenge to the existing theory of host-parasite interactions: the discovery of bacterial endosymbionts that increase their hosts' resistance to parasites [14]–[17]. When a symbiont protects a host against a parasite, one has to consider the coevolution of three, not two, species, with all the resulting complications. In particular, it is now important to distinguish between host-parasite and symbiont-parasite genetic interactions. It is for example possible for one to be of the GFG, and the other of the MA kind. Indeed, specialisation of symbiont strains to parasite genotypes and apparent lack of host-parasite specialisation characterises the protection against parasitoid wasps that the endosymbiotic bacterium *Hamiltonella defensa* lends to aphids [18], [19]. Another important aspect that models of such systems should address is the spread of symbionts in, and loss from, the host populations, and the coevolutionary impact of these processes.

In this paper we build a generic model of the coevolution of hosts, symbionts and parasites. We incorporate as independent, tunable parameters the strength of the reciprocal selection acting on hosts and parasites, the fitness penalty for harbouring symbionts, the efficiency of horizontal and vertical transmission of symbionts, and the genetic interactions among all players. In a straightforward extension of the standard approach, the last factor is subsumed in a real-valued matrix, and we randomly sample many such matrices and simulate our model for each. In this way we are able to cover a range of potential host-symbiont-parasite systems and, importantly, decouple the effects of genetic specialisation patterns from the other factors. In addition, we analyse separately a collection of matrices describing protective symbionts acting within the established MA and GFG frameworks. Throughout, we do not treat genetic specialisation as a binary property; instead, we devise a numerical index of specificity. We find that specificity as defined in this paper strongly influences important coevolutionary outcomes of the models, such as the genotype frequency dynamics, maintenance of allelic diversity and mean host and parasite fitness. Our simulations show that these characteristics depend also on symbiont-related processes, especially the reliability of their maternal inheritance.

## Methods

### Master matrix

We assume that hosts and parasites reproduce asexually and consider one haploid locus and two alleles in each of the three protagonists: host, symbiont and parasite; we also model hosts without symbionts. This gives rise to two parasite genotypes: 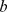 and 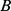, and six combined host-symbiont genotypes or *associations*: 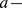, 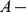, 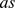, 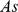, 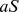 and 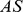. The blank “
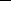
” denotes absence of symbiont, and so the symbiont-free hosts 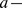 and 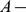 are also formally considered to be associations. We subsume any individual interaction pattern of the host-symbiont and parasite genotypes in a 6
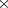
2 *master matrix* denoted 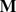. Each entry in this matrix falls in the 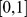 interval and is interpreted as the degree of resistance of the particular host-symbiont association to the particular parasite genotype. To give a concrete example, if 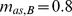, then every 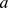 host carrying the 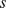 symbiont will suffer only 20% of the maximal potential fitness damage from the 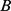 parasite. For the majority of the analyses we rely on random generation of such matrices in order to cover a wide range of possible host-parasite relationships (see “Model sampling and simulation”).

**Figure 1:**
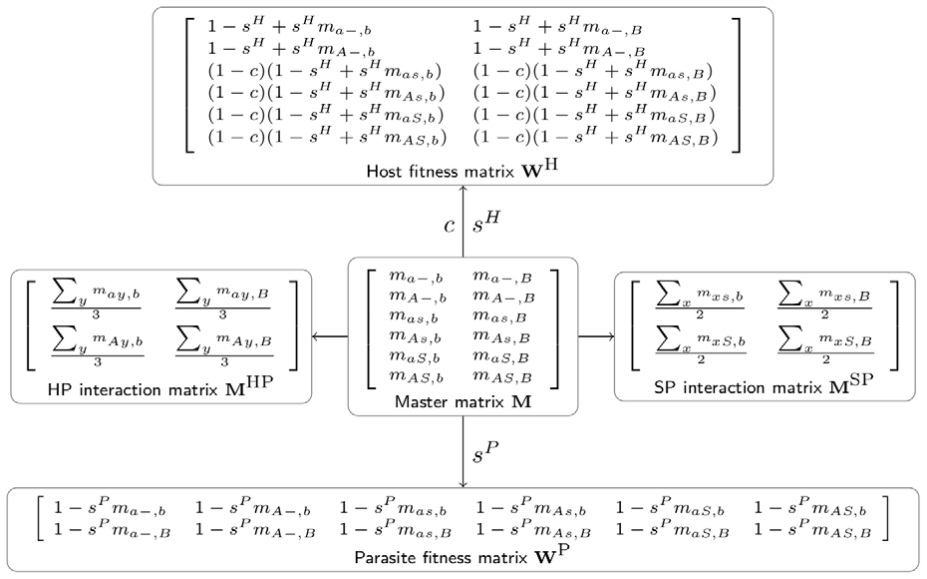
The relationship between the master matrix and the four auxiliary matrices.

We use three additional parameters in our coevolutionary setup: the maximum strength of selection that the parasites can exert on hosts 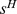 (e.g. 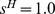 means the host can have zero fitness as a result of infection, that is be sterilised or killed before it reproduces), the corresponding parameter 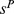 representing the strength of selection on parasites (which can be interpreted as the maximal fitness penalty for failing to infect a host), and the fitness penalty 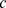 the hosts pay for harbouring protective symbionts; see Table 1 for an overview of parameters and their values. These parameters are used to derive the host and parasite fitness matrices, 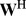 and 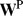 respectively, from the master matrix 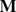 as follows (see also Figure 1):
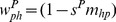
(1)

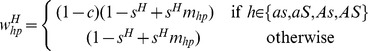
(2)(Here, and throughout the paper, we use lowercase letters to refer to the entries of the matrix denoted by the corresponding uppercase letter.) Each entry of the fitness matrix specifies the relative fitness consequence of the interaction between a particular host-symbiont association and a particular parasite genotype. Again, to give a concrete example, 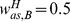 means that the fitness of the host-symbiont association 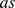 when faced with the parasite genotype 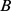 is half that of a symbiont-free uninfected host. By definition, host and parasite fitness values are fully anti-correlated in our model, reflecting the antagonistic nature of host-parasite relationships (but see also [13]).

**Table 1:** Parameters of the model.

parameter	meaning	values used
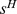	maximal selection on host	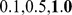
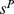	maximal selection on parasite	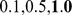
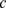	cost of harbouring symbionts	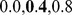
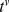	reliability of vertical transmission of symbionts	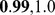
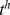	rate of horizontal transmission of symbionts	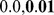

Parameters of the model. The values in bold roughly correspond to the *Hamiltonella defensa*-mediated aphid-wasp systems [14], [37].

### Specificity

Instead of generalising the concept of genetic specialisation to three species, we prefer to analyse the specificity of genetic interactions between pairs of species separately. To this end we transform the master matrix into three 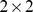 matrices 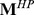, 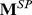 and 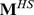, each time averaging out the contribution of one species (symbiont, host and parasite, respectively). Thus, each of these matrices serves as a proxy for the genetic interaction of the remaining two species. Formally, they are defined by:
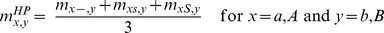
(3)

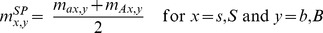
(4)

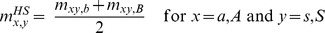
(5)


In this paper we focus on 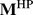 and 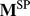. Figure S1 contains the corresponding results for 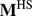.

Our basic assumption in formalisation of specificity is that a relationship between two coevolving species 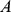 and 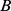 is specific if there are two genotypes of the 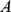 species, say 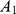 and 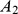, and two of the 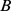 species, 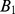 and 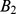, such that 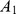 is better adapted than 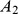 to 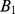, but 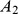 is better adapted than 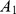 to 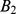
—or analogously with 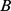
s and 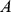
s swapped around. This is the same as saying that there is a genotype
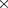
genotype interaction between 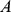 and 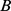, or that the reaction norms for two 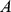 or two 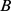 genotypes cross. This definition is also easily expressible in terms of interaction matrices. Taking the 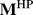 as an example, we say that the interaction it subsumes is specific if and only if 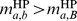 but 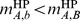, or this condition holds with 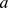/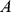 or 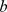/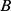 switched in a consistent manner. When 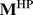 is specific, we define its index of specificity, 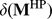, to be the minimal additive disturbance necessary to bring 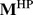 into a non-specific form. Formally:
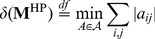
(6)where 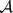 is the set of matrices such that 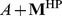 is non-specific.

The above definition can be generalised to cover arbitrary 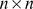 and 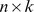 matrices. These constructions are described in Text S1. In the remainder of the paper we are only concerned with the specificity of 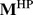 and 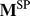. For these, and for any 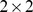 matrices in general, observe that 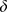 ranges between 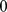 and 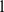, with 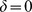 only for non-specific matrices such as the GFG matrix 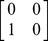, and 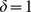 only for the MA matrix 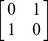 and the Inverse Matching Alleles matrix 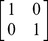. The IMA model [20] assumes that the host is *resistant* if and only if it can match all parasite alleles; in the 2
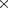
2 case it is formally equivalent to the MA model because their matrices are mirror images of each other. For square 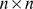 matrices with 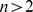, the GFG and IMA matrices acquire intermediate specificity, while the MA matrix remains the most specific.

### Model sampling and simulation

**Figure 2:**
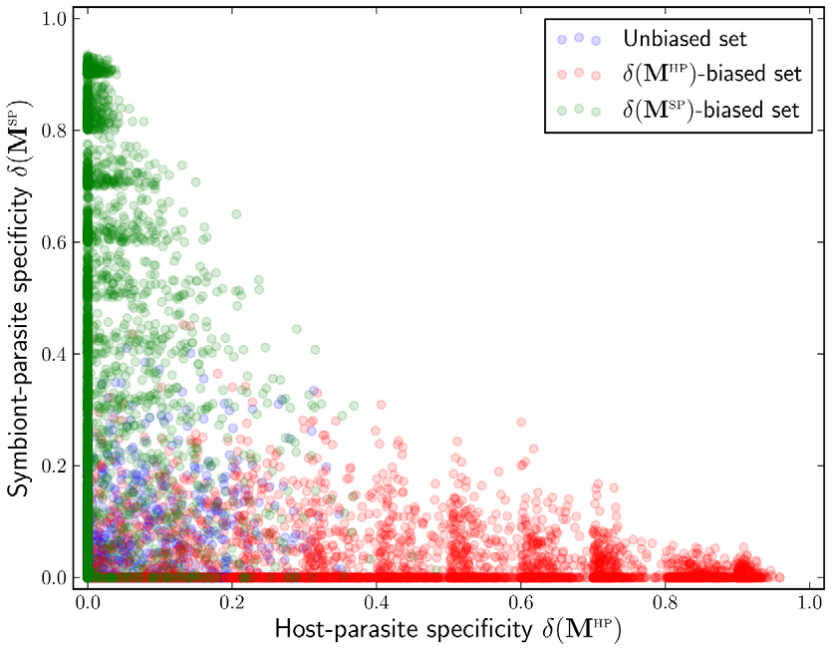
Distribution of host-parasite and symbiont-parasite specificities in the two sets of master matrices analysed in this study (red and green). A set of 4000 master matrices generated by independent sampling of entries from an uniform distribution on [0,1] is provided for comparison (blue).

With the exception of the MA- and GFG-based host-symbiont-parasite relationships analysed in “Protective symbionts in the MA and GFG frameworks”, we kept the range of investigated relationships as broad as possible by generating a large number of random master matrices. Because we are interested in the effects of specificity, our goal was to have two collections of matrices, each uniformly distributed with respect to one of the specificity scores. Ideally, one would generate enough matrices by sampling the entries independently from the uniform distribution (i.u.d.) on [0,1], and then select the two matrix collections from this sample. Unfortunately, i.u.d. sampling yields no high-specificity matrices in reasonable time, because their entries have extreme values and are highly dependent on each other (see Text S2). To overcome this problem, we separated the [0,1] interval into ten non-overlapping sub-intervals of length 0.1, and for each sub-interval we randomly generated master matrices until we had 400 with the specificity score falling in it. For intervals up to [0.6,0.7] the matrices were obtained by i.u.d. sampling. For the remaining three the matrices were independently sampled from a symmetric bimodal distribution with modes 0 and 1 (probability density of 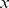 being 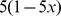 for 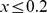, 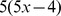 for 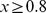, and 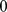 otherwise), ensuring polarised matrix entries, which is a characteristic property of high-specificity matrices. Of all matrices we further required that the symbionts do not impair host resistance, which translates into the simple criterion: 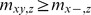 for all 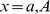, 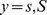 and 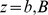. We performed this procedure twice, once for HP-specificity and once for SP, obtaining two sets of 4000 matrices distributed in an approximately uniform fashion with respect to 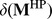 and 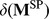; see Figure 2.

We considered three values of each of the three supplementary parameters, 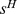, 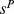 and 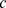 (see Table 1 for an overview of parameters), thus deriving 27 pairs of fitness matrices for each master matrix (see Figure 1). For each pair of fitness matrices we simulated 20000 generations of coevolution, the first 10000 of which were considered the burn-in period and discarded. We worked with infinite population sizes, meaning that we tracked frequencies of host-symbiont and parasite genotypes rather than population sizes. At each generation the symbionts colonised the symbiont-free hosts at the mass-action basal rate 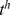, then selection was allowed to operate, and finally the constant fraction of 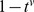 of symbiont-harbouring hosts lost the symbionts. The selection step was performed as follows (see also [13]): assume that 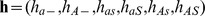 is the vector of host-symbiont association frequencies, and 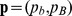 the vector of parasite genotype frequencies. Then the post-selection frequencies 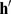 and 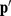 are given by:
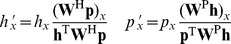
The numerator in the above expressions gives the fitness of genotype 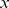, obtained as the sum of the 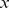
th row of the fitness matrix entries, weighed by the frequencies of the genotypes of the antagonists. The underlying assumption is that the more common a particular opponent is, the more the performance against it contributes to fitness. The denominator, which is independent of 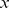, is the mean host or parasite fitness and ensures that the frequencies add up to one after selection.

For each master matrix we simulated and analysed 108 models, because there are 108 auxiliary parameter combinations (Table 1). Each model was first simulated for 10 randomly chosen starting frequency vectors, and then once more for equal starting frequencies of all genotypes, with the results of the last run used for evaluation. Approximately 10% of the models were ambiguous, in that the results of the 11 simulations were not consistent with each other. We also had trivial models: whenever 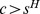, the symbiont-bearing hosts inevitably went extinct, since the cost of symbionts exceeded any damage the parasites could inflict and we assumed the horizontal transfer to be rare. Unless noted otherwise, the ambiguous models are included in the analyses that follow, but the trivial ones are not.

## Results

### Protective symbionts in the MA and GFG models

We begin by considering two simple models incorporating symbiotic protection into the Gene For Gene and Matching Alleles frameworks. We assume that the hosts' innate resistance to the parasites follows one of these two classic principles, but the symbionts may confer a partial degree of resistance 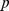 to the non-resistant hosts. We further assume that the symbiotic protection is specialised: symbiont 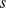 only protects against the parasite 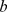, and 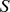 against 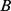. This leads to two master matrices: 
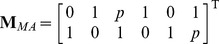
 and 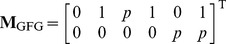. Setting 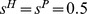, and remembering that harbouring symbionts (regardless of whether they are needed for protection) comes at the cost 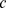, we derived two pairs of host and parasite fitness matrices using equations (1) and (2). We then simulated the two models for different combinations of 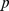 and 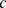, under perfect maternal inheritance of symbionts (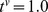) and without horizontal transfer events (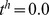).

We found that despite the presence of symbionts, the dynamical behaviour of the models is similar to that of their classic symbiont-free counterparts. All MA models exhibit strong oscillations of host and parasite allele frequencies, while in the GFG models the allele frequencies stabilise. The balance of cost and protection quality determines the fate of symbionts in both models. In the MA model, the symbionts become fixed in the population if 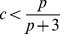; otherwise they go extinct. The corresponding condition for the GFG model, 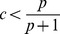, is less strict because the symbiont-provided protection is essential against the parasite 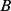; for the same reason the symbiont 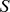 is more common than 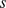 in this model. These formulae can be derived using the definition of the host fitness matrix and considering when the symbionts confer a net fitness benefit to their hosts, and were corroborated by the simulations.

We now give an intuitive specificity-based interpretation of these results. The host-parasite specificity is high in the MA models: 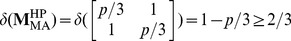, and therefore the negative frequency-dependent selection drives the oscillations of host and parasite alleles easily regardless of whether the symbionts are present. In the GFG models the host-parasite specificity is zero: 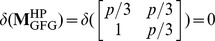, and thus in the absence of symbionts the allele frequencies are stable. When the symbionts are present, the moderate symbiont-parasite specificity for high 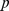 (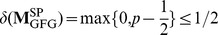) could be expected to result in oscillations (see “Allelic diversity and frequency dynamics”). However, in these models there is also the host-symbiont association 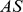 that nullifies this effect of specificity because it is more resistant than any other to both parasites. In the remainder of the paper we substantiate and expand these intuitions by linking specificities to coevolutionary outcomes of models based on randomly generated master matrices.

### Cycling and the fate of host-symbiont associations

**Figure 3:**
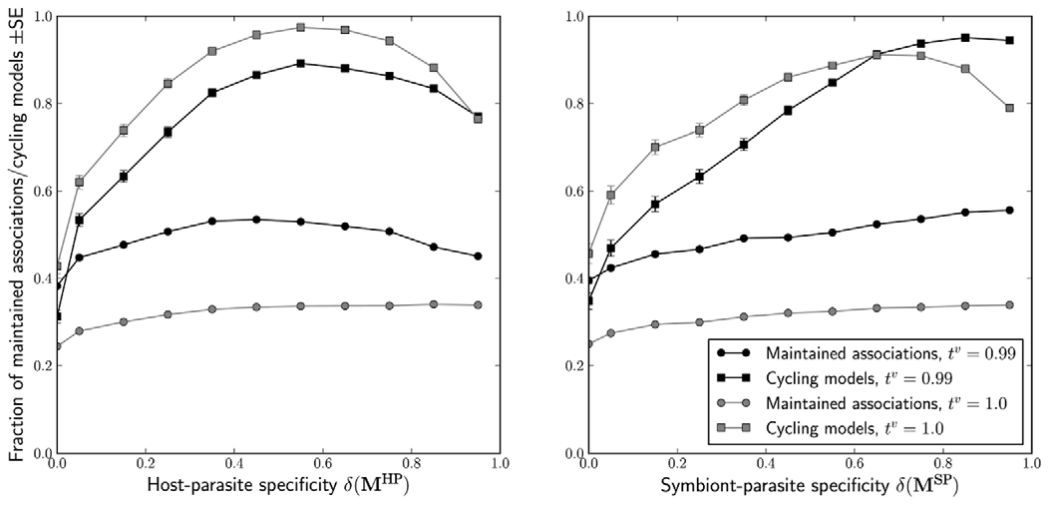
Impact of specificity and symbiont inheritance rate on the survival and frequency dynamics of host-symbiont associations. In both graphs, as well as in Figures 4 and 6, the master matrices are divided into 11 bins according to their appropriate specificity. The first bin holds exclusively the non-specific matrices, and the remaining 10 are equally spaced across [0,1]. All bins hold similar numbers of matrices (see Figure 2).

In classical models incorporating genetic specialisation such as the MA model, pronounced oscillations of genotype frequencies often ensure indefinite maintenance of at least two genotypes. Here, we examine to what extent these effects reappear in our three-species setup. We say that a model cycles if the frequency of at least one host-symbiont association has six or more local extrema over the assessment period, and the amplitude between the extrema does not decrease; also, we declare an association lost from the population if its mean frequency over the assessment period is less than 10^−3^. We found that while both the host-parasite and symbionts-parasite specificities broadly promote cycling and diversity (Figure 3), they do so in qualitatively different ways. For HP specificity, the prevalence of cycling and the mean number of host-symbiont associations maintained in the model reach maximum values for master matrices with intermediate values of 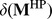. For SP-specificity, both measures increase across the entire range of 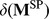, with one exception (see below). In both cases, models based on non-specific matrices maintain the fewest associations on average and are the least likely to cycle.

The fidelity of vertical transmission of symbionts has a strong quantitative effect on the fate of host-symbiont associations and on their dynamics. Perfect symbiont inheritance (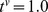) is often necessary to maintain symbiont infections, but it can also lead to the extinction of the symbiont-free hosts 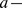 and 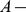, as these populations often depend on the influx of hosts from the infected lineages (see also [21]). Fewer models oscillate when 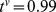 than when 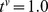, likely due to the interference of the symbiont-free hosts with the frequency-dependent selection driving the cycles. This hypothesis is consistent with the effect disappearing for master matrices with extreme 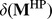, where the host-parasite relationship becomes determined by host and parasite alleles only, and no significant interference is possible from hosts differing only in their symbiont infection state. For high values of 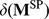 on the other hand, we found that the trend is reversed and perfect vertical transmission of symbionts results in less frequent cycling. Here, the probable explanation is that the symbiont-free hosts cannot engage in cycling because they necessarily go extinct unless they are replenished via symbiont loss (see Text S2 for more on matrices of high specificity).

The number of maintained associations and the prevalence of cycling are virtually the same for models differing only in the presence of weak horizontal transmission of symbionts (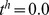 or 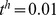).

### Allelic diversity and frequency dynamics

**Figure 4:**
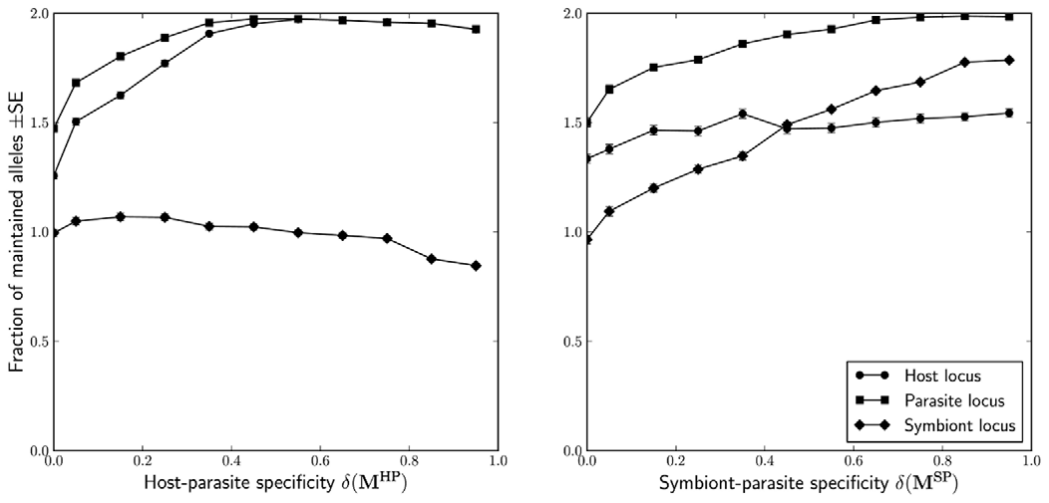
Specificity and maintenance of allelic diversity. Note that the model is constructed is such a way that both symbiont alleles but no more than one host and one parasite allele can be lost.

We turn now to the question of allelic diversity, that is the maintenance of the individual host, symbiont and parasite alleles. We found that the genetic specialisation between antagonist species strongly promotes allelic diversity in these species (Figure 4). Moderate and high host-parasite specificity all but guaranteed the survival of both host and both parasite alleles. We found a similar effect of 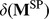 on symbiont and parasite alleles, but the mean symbiont allele diversity taken across all analysed models increases considerably more slowly due to the cost associated with harbouring symbionts that is built into most of these models (see Methods). There is no effect of the specialisation on the allelic diversity in the non-specialised species, that is 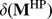 and 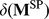 do not influence the likelihood of loss of symbiont and host alleles, respectively. Again, we found no impact of the horizontal transmission of symbionts on the allelic diversity in any of the three species.

**Figure 5:**
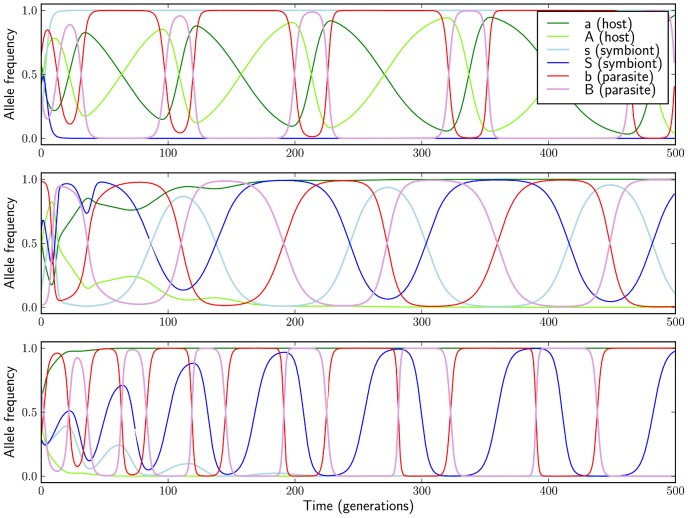
Symbionts may take part in Red Queen dynamics. Top: “classical” RQ dynamics, with frequencies of host and parasite alleles oscillating due to antagonistic coevolution between the two species (symbiont allele frequencies remain stable). Middle: symbiont and parasite alleles oscillate, and the host background is stable. Bottom: host background is stable, one symbiont allele is lost, and the frequency of the other oscillates with the frequencies of the two parasite alleles. All simulations performed with random initial frequencies, and for parameters values shown in bold in Table 1, except 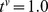.

The presence of symbionts results in novel kinds of Red Queen dynamics (Figure 5). Traditionally, this term refers to persistent oscillations of both host and parasite genotype frequencies driven by the genetic composition of the antagonist population (also known as negative frequency-dependence). Under considerable symbiont-parasite specificity, oscillations of symbiont allele frequencies may replace those of the host alleles. The result is a dynamical pattern that would be regarded as cryptic if one were unaware of the existence of symbionts: the host allele frequencies remain stable but those of the parasite oscillate. Another possibility is that one symbiont allele is lost, but the other periodically rises and falls in frequency in the host population due to its specialisation to one of the parasite alleles.

### Mean host and parasite fitness

**Figure 6:**
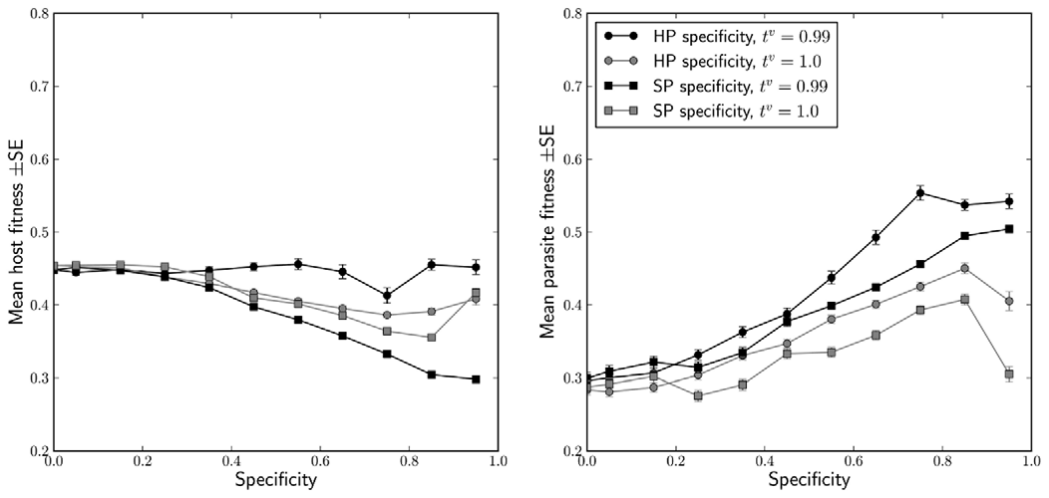
The influence of host-parasite and symbiont-parasite specificities on host and parasite population fitness, and its interaction with the fidelity of symbiont inheritance. The results are presented for the values of parameters roughly corresponding to the aphid-wasp system [14], [37], but qualitatively similar patterns were observed for most of the other parameter combinations.

Lastly, we investigated the dependence of mean host and parasite fitnesses on the host-parasite and symbiont-parasite specificities, and on the parameters of the model. Mean fitness within the host population can be regarded as a measure of how well the hosts are adapted on average to resist infection by the parasites, and analogously for the parasites and their mean fitness. As a general rule, mean parasite fitness increases and mean host fitness decreases with increasing specificity (see Figure 6). From the host population's perspective this effect can be attributed to the specificity widening the gap between the fitnesses of well-adapted and maladapted associations (see Text S2), and the contribution of the latter to the mean fitness in the presence of efficient parasites. However, as the entries of fitness matrices become more and more polarised for high or extreme specificities, the selection against the maladapted associations becomes more and more swift. Consequently they cease to contribute to the mean fitness and the trend is halted or even reversed.

The fidelity of symbiont inheritance plays a similar role to that discussed in “Cycling and the fate of host-symbiont associations”. Imperfect inheritance maintains a small yet stable population of symbiont-free hosts even when they are severely selectively disadvantaged, e.g. for high values of 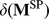, and the mean host fitness is reduced. On the other hand, when host-parasite specificity is high, symbiont-free hosts enjoy protection similar to that of their symbiont-bearing counterparts but do not pay the fitness penalty for symbiosis, and thus their influx increases the mean host fitness. We found no influence of horizontal transmission of symbionts on the mean fitness of either species throughout our analyses.

We observed the mean host fitness decrease with increasing strength of selection the hosts are under (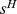) and the costs of symbiont protection (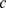). Similarly, mean parasite fitness is sensitive to 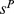, with stronger selection leading to lower fitness. These findings were entirely expected, since high values of 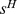, 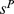 and 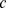 make for low average entries in the fitness matrices. The antagonistic nature of the relationship was visible in that the increases of mean host fitness generally coincided with decreases of mean parasite fitness, and vice versa. This too can be traced back to fitness matrices, more precisely to the anti-correlation of 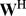 and 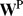.

## Discussion

Genetic specialisation has been recognised in the literature on host-parasite interactions as a fundamental concept in its own right [22]. To the best of our knowledge, we provided here the first method of quantifying it in the context of coevolutionary modelling. By basing the construction on the concept of fitness matrices central to host-parasite theory [23], [24], we ensured that our method is applicable to a wide range of models, extant and future. For actual biological systems for which fitness matrices can be approximated experimentally, for example in factorial experiments, our index can be used directly to generate concrete predictions about the coevolutionary dynamics and genetic diversity.

Our work was inspired by that of Agrawal and Lively [12], who analysed host-parasite coevolutionary dynamics for a range of non-standard fitness matrices. Their setup was based around a single parameter 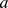 so that 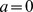 yielded the MA model, 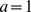 the GFG, and intermediate values gave non-standard matrices. However, Agrawal and Lively's 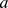 is not an extrinsic measure of a matrix, and therefore an independent characterisation of a relationship, but an intrinsic assumption used to construct it. In particular, if costs of resistance and virulence and the selection strength are fixed, the value of 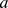 determines the matrix. Thus, our work is more general because it makes it possible to talk about the specificity of arbitrary matrices, and because we base our conclusions on a much wider range of models. In these respects it resembles a study of Engelstädter and Bonhoeffer [13], where antagonicity, another property of arbitrary fitness matrices, was developed to analyse coevolutionary dynamics.

Our specificity index is related to the notions of nestedness and matrix temperature introduced by Atmar and Patterson [25] to study the extinction of species in fragmented habitats, later used for structural analysis of plant-animal interaction networks [26], [27]. In this approach, one starts with a presence-absence matrix, that is a binary matrix where 1 denotes an existing plant-animal interaction, or the presence of a species in a habitat, and 0 the absence thereof. The matrix is rearranged so that the rows and columns corresponding to more generalist species or more hospitable habitats appear higher (rows) and farther to the left (columns). The matrix temperature is then obtained by penalising deviations of this rearranged matrix from the fully nested matrix, where ones appear only above a generalised diagonal and zeroes only below. A fully nested matrix is non-specific by our definition, but many non-specific matrices are not fully nested. Hence, matrices of high specificity will tend to have high temperature, but temperature may be different for matrices of the same specificity and vice versa. Importantly, specificity is defined for arbitrary matrices while temperature applies to binary matrices only. The similarity of the two measures suggests nevertheless that they capture facets of an essential property of biological interaction networks, and therefore that our specificity index may be applicable more widely than only to host-parasite coevolution.

Our setup explicitly included a heritable symbiotic species increasing the hosts' resistance to the parasites. Such beneficial symbionts can play fundamental roles in the the ecology and evolution of their hosts, highlighting the need for comprehensive treatment of the forces shaping symbionts' own spread and evolution [28]. Defensive symbionts are transmitted from mother to offspring with very high fidelity, with the link between protection and maternal transmission also strongly supported by theory [29]–[31], but lateral transfer appears to be relatively rare on the ecological timescale [32], [33]. Accordingly, we analysed the impact of occasional vertical loss and occasional horizontal transfer of symbionts on the coevolution of hosts and parasites. We found that a small population of symbiont-free hosts maintained exclusively by the sporadic failure of vertical transmission can disrupt the Red Queen dynamics driven by the specialisation of parasite alleles and host-symbiont associations. Sporadic lateral transfer had no effect on the results of simulations, but given the well-documented role of lateral transfer in the interspecific spread of bacterial symbionts [34], [35], we believe that better estimates of the basal rate of transfer (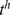) ought to be obtained before discounting its role in the coevolutionary dynamics of the three interacting species. Still, it seems plausible to us that horizontal transfer is important in establishing the initial symbiont infections, but not in their subsequent fate in the host populations, which is governed mainly by the cost-benefit trade-off.

The inclusion of protective symbionts as the third species highlighted the interplay of coevolutionary antagonicity and specificity. The genetic specificity between antagonist species had stronger effects on the vital properties of the system such as cycling and maintenance of alleles than the specificity of the mutualist host-symbiont relationship (Figure S1). This result dovetails with that of Engelstädter and Bonhoeffer [13], who showed that antagonicity of interaction promotes allelic diversity. We wish to point out, however, that our model is simplistic in two important respects. First, it ignores the fact that in addition to providing protection from parasites and pathogens, maternally transmitted symbionts may also manipulate the host reproductive phenotype in various ways (reviewed by Engelstädter and Hurst [36]), and thus the relationship between the host and the symbiont may be less mutualistic than envisaged here. Second, our model is fully deterministic, and as such it does not incorporate genetic drift. However, when the genotypes of two antagonist species are highly specialised to each other but not to the third one, drift can be expected to play a significant role in the evolution of the latter.

Our work may have interesting implications for the Red Queen Hypothesis (RQH)—the idea that host-parasite coevolution underlies the evolution of sex and recombination [1], [4], [24]. We assumed in our model that hosts reproduce asexually. As a consequence, defensive symbionts and host resistance genes are predominantly co-inherited, except for horizontal transmission events that we assumed to be rare. This lack of recombination, in tandem with the strong epistatic interactions between the symbionts and the nuclear genes that are implicit in many of our master matrices, can create pronounced fluctuations of the linkage disequilibrium (LD) between the nuclear locus and the symbiont “locus” when diversity is maintained at both loci (results not shown). In conventional Red Queen models considering only nuclear loci, such LD fluctuations are a prerequisite for recombination modifiers to be under positive selection. In our model, sexual reproduction would entail free recombination between the nuclear and the symbiont locus due to their different modes of inheritance (Mendelian vs. maternal). Therefore, we speculate that modifier alleles inducing sexual reproduction may be selected for under some of our host-symbiont-parasite interaction matrices. This tripartite version of the Red Queen represents an exciting avenue for future research.

## Supporting Information

Figure S1 **Host-symbiont specificity.** The influence of host-symbiont specificity on the diversity of host-symbiont associations, likelihood of cycling, allelic diversity, and mean host and parasite fitnesses.
(TIFF)

Text S1 **Specificity of arbitrary matrices.** This appendix shows how to generalise the definition of specificity introduced in the main text to operate on matrices of arbitrary size and shape.
(PDF)

Text S2 **Matrices of high specificity.** This appendix discusses the properties of high-specificity matrices.
(PDF)
