# Supplementary figures and images for: On Genetic Specificity in Symbiont-Mediated Host-Parasite Coevolution
Source: PLoS Comput Biol. 2012 Aug 30;8(8):e1002633. doi: 10.1371/journal.pcbi.1002633 (PMC3431304; doi:10.1371/journal.pcbi.1002633)

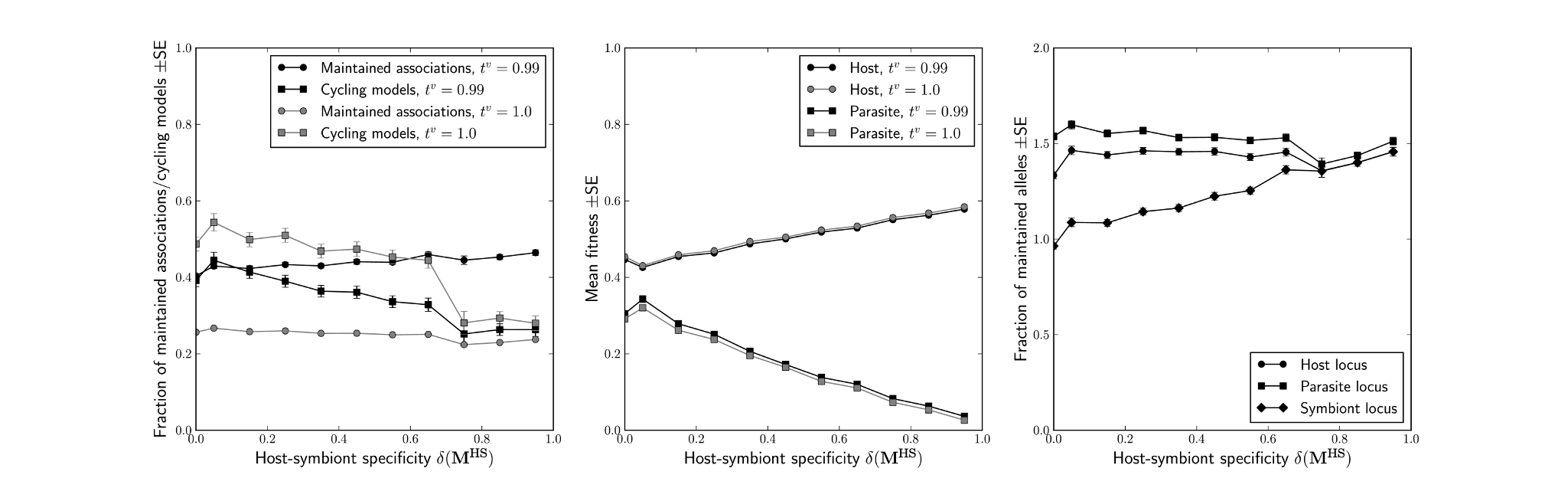

Supplement: Figure S1 — Host-symbiont specificity. The influence of host-symbiont specificity on the diversity of host-symbiont associations, likelihood of cycling, allelic diversity, and mean host and parasite fitnesses. (TIFF) [file pcbi.1002633.s001.tiff]
