# Supplementary material for: On Genetic Specificity in Symbiont-Mediated Host-Parasite Coevolution
Source: PLoS Comput Biol. 2012 Aug 30;8(8):e1002633. doi: 10.1371/journal.pcbi.1002633 (PMC3431304; doi:10.1371/journal.pcbi.1002633)
Supplement: Text S1 — Specificity of arbitrary matrices. This appendix shows how to generalise the definition of specificity introduced in the main text to operate on matrices of arbitrary size and shape. (PDF) [file pcbi.1002633.s002.pdf]

# S1. Specificity of arbitrary matrices

The specificity index, $\delta$, can be generalised to operate on arbitrary matrices. The generalisation works best for square matrices, however, and so we focus on these.

Recall that we defined an interaction between the genotypes of two species to be specific if there are two $A$-genotypes, $A_1$ and $A_2$, and two $B$-genotypes, $B_1$ and $B_2$, such that $A_1$ is better adapted than $A_2$ to $B_1$ but $A_2$ is better adapted than $A_1$ to $B_2$, or analogously for two $B$-genotypes (see "Specificity" in the main text). The converse of this condition is thus the definition of a non-specific interaction. For the case of $n$ genotypes it reads: there exist an ordering of $A$-genotypes $a_1 < \ldots < a_n$ (or $b_1 < \ldots < b_n$ of $B$-genotypes) such that for every $i$, $a_{i+1}$ is better or equally adapted than $a_i$ to all $b_j$s (or analogously if the postulated order is on $B$-genotypes). In other words, one of the two species has the property that for any pair of its genotypes, one is at least as well adapted as the other to all genotypes of the other species.

Now let us suppose that the interaction of the two species is subsumed in an $n \times n$ matrix $M$—a master matrix, or a fitness matrix, or similar. In this approach, genotypes correspond to rows and columns of the matrix. The matrix is thus non-specific is there is an appropriate ordering $\sigma_1 \ldots \sigma_n$ of its rows, such that for all $i$ and $j$, $m_{\sigma_{i+1},j} > m_{\sigma_i,j}$ (or the analogous condition holds for the columns). Finally, denoting by $\mathcal{A}$ the set of $n \times n$ matrices $A$ such that $M + A$ is non-specific, we can define the specificity of $M$ by:

$$\delta(M) \stackrel{\text{df}}{=} \frac{2}{n^2 - n} \min_{A \in \mathcal{A}} \sum_{i,j} |a_{ij}|$$

As before, the expression $\min_{A\in\mathcal{A}} \sum_{i,j} |a_{ij}|$ denotes the minimal additive disturbance needed to bring $M$ to a non-specific form. The scaling factor $\frac{2}{n^2-n}$ ensures that the values of $\delta(M)$ fall between 0 and 1. To see that, observe that the disturbance required to bring the maximally specific $n \times n$ matrix—the MA matrix—to a non-specific form is precisely $\frac{n^2-n}{2}$: the relevant $A$ matrix has ones above the diagonal and zeroes everywhere else.

When the interaction model is given by two partially or fully independent matrices, e.g. when the relationship if not fully antagonistic and a fitness matrix is given for each of the two species separately, the smaller of the two specificities should be taken to characterise the interaction. Lastly, when the matrix is rectangular, the formula above is applicable as well, but the scaling factor cannot ensure anymore that the range of specificities is precisely $[0, 1]$.
