# Supplementary material for: On Genetic Specificity in Symbiont-Mediated Host-Parasite Coevolution
Source: PLoS Comput Biol. 2012 Aug 30;8(8):e1002633. doi: 10.1371/journal.pcbi.1002633 (PMC3431304; doi:10.1371/journal.pcbi.1002633)
Supplement: Text S2 — Matrices of high specificity. This appendix discusses the properties of high-specificity matrices. (PDF) [file pcbi.1002633.s003.pdf]

S2. Matrices of high specificity

Master matrices of high specificity of any of the three kinds are of particular forms: the higher the specificity, the closer the matrix is to one of the limit matrices in Figure 1 below. These matrices are obtained by solving the equations $\mathbf{M}^{\mathrm{HP}} = \left(\begin{smallmatrix}1 & 0\\ 0 & 1\end{smallmatrix}\right)$, $\mathbf{M}^{\mathrm{SP}} = \left(\begin{smallmatrix}1 & 0\\ 0 & 1\end{smallmatrix}\right)$, and $\mathbf{M}^{\mathrm{HS}} = \left(\begin{smallmatrix}1 & 0\\ 0 & 1\end{smallmatrix}\right)$, under the constraint that the symbionts are in principle beneficial, i.e. $m_{xy,z} \geq m_{x-,z}$ for all $x = a, A$, $y = s, S$ and $z = b, B$. Below we briefly discuss the consequences of high specificities.

High HP-specificity reduces the differences between symbiont-bearing and symbiont-free hosts when the host and parasite background is fixed: $|m_{xy,z} - m_{x-,z}|$ becomes close to zero. Thus it can be expected that the persistence of symbionts becomes more sensitive to the costs of infection. High host-parasite specificity also reduces the differences between the effects of different symbiont alleles.

High SP-specificity reduces the effect of the host allele on the outcome of the interaction: $|m_{ay,z} - m_{Ay,z}|$ becomes close to zero. Another consequence of high SP-specificity is that the symbiont-free hosts are not able to resist infection.

High HS-specificity makes the outcome of the parasite attack insensitive to the parasite genotype: $|m_{xy,b} - m_{xy,B}|$ is close to zero. Symbiont-free hosts are very susceptible to infection, but two of the four host-symbiont associations are largely immune.

$$
\begin{array}{c}\\ \text{a-}\\ \text{A-}\\ \text{as}\\ \text{As}\\ \text{aS}\\ \text{AS}\end{array}
\begin{array}{c}\begin{array}{cc}\text{b} & \text{B}\end{array}\\ \begin{pmatrix}1 & 0\\ 0 & 1\\ 1 & 0\\ 0 & 1\\ 1 & 0\\ 0 & 1\end{pmatrix}\end{array}
\qquad
\begin{array}{c}\\ \text{a-}\\ \text{A-}\\ \text{as}\\ \text{As}\\ \text{aS}\\ \text{AS}\end{array}
\begin{array}{c}\begin{array}{cc}\text{b} & \text{B}\end{array}\\ \begin{pmatrix}0 & 0\\ 0 & 0\\ 1 & 0\\ 1 & 0\\ 0 & 1\\ 0 & 1\end{pmatrix}\end{array}
\qquad
\begin{array}{c}\\ \text{a-}\\ \text{A-}\\ \text{as}\\ \text{As}\\ \text{aS}\\ \text{AS}\end{array}
\begin{array}{c}\begin{array}{cc}\text{b} & \text{B}\end{array}\\ \begin{pmatrix}0 & 0\\ 0 & 0\\ 1 & 1\\ 0 & 0\\ 0 & 0\\ 1 & 1\end{pmatrix}\end{array}
$$

Figure 1: Master matrices with maximal host-parasite (l), symbiont-parasite (m), and host-symbiont (r) specificities. Each matrix has a symmetric version with the same property.
